# Supplementary material for: Generation of Brain Microvascular Endothelial-like Cells from Human iPS Cell-Derived Endothelial Progenitor Cells Using TGF-β Receptor Inhibitor, Laminin 511 Fragment, and Neuronal Cell Culture Supplements
Source: Pharmaceutics. 2022 Dec 2;14(12):2697. doi: 10.3390/pharmaceutics14122697 (PMC9785586; doi:10.3390/pharmaceutics14122697)
Supplement: Supplementary file 1 [file pharmaceutics-14-02697-s001.zip › pharmaceutics-2045697-supplementary.pdf]

Additional file S1

Generation of Brain Microvascular Endothelial-like Cells from Human iPS Cell-Derived Endothelial Progenitor Cells Using TGF-β Receptor Inhibitor, Laminin 511 Fragment, and Neuronal Cell Culture Supplements

Hiromasa Aoki, Misaki Yamashita, Tadahiro Hashita, Takahiro Iwao, Mineyoshi Aoyama and Tamihide Matsunaga

Components

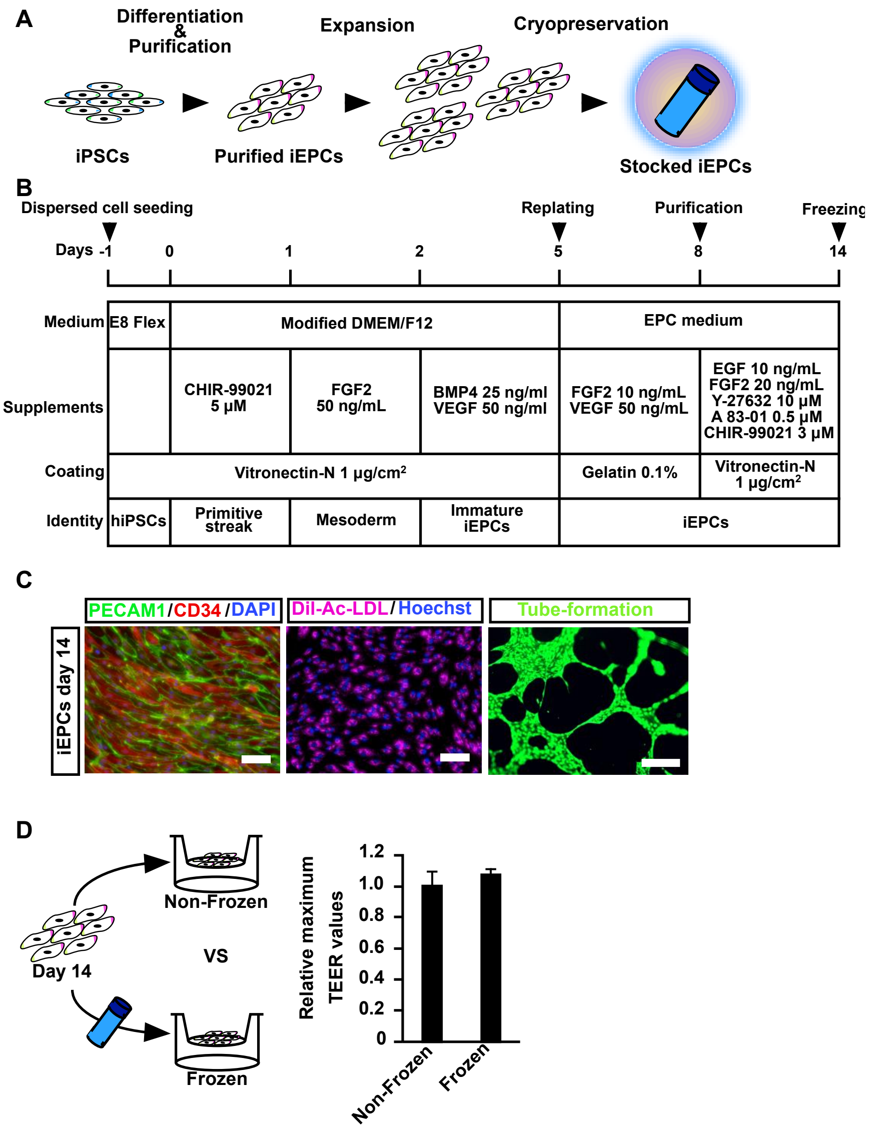

Figure S1. Effect of freeze–thawing on the physical barrier function of iEPCs
(A and B) Scheme for the differentiation of iEPCs. (C) Immunofluorescence analyses of PECAM1 (green), CD34 (red), and Dil-Ac-LDL uptake and tube formation assays of 610B1-derived iEPCs. DAPI = blue (immunofluorescence analyses); Hoechst 33342 (Dil-Ac-LDL uptake assay) = blue; Dil-Ac-LDL = pink; calcein = green. Scale bars = 100 µm (immunofluorescence analyses and Dil-Ac-LDL uptake assay) and 500 µm (tube formation assay). (D) Measurement of TEER values of frozen and non-frozen iEPCs (7×10^5 cells/insert) on day 14. Data are presented as the mean ± SD ($n = 3$; Student's t-test).

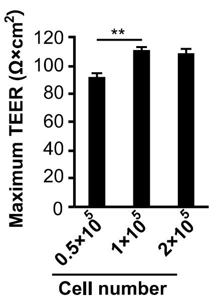

Figure S2. Effect of seeding cell number on TEER values

Thawed iEPCs were seeded at 0.5 × 10^5, 1× 10^5, and 2 × 10^5 cells/insert and cultured in HE-SFM supplemented with 20 ng/mL FGF2, penicillin–streptomycin solution, 7.5% B-27 supplement, and 1 µM A 83-01 on cell culture inserts coated with a coating solution consisting of fibronectin, collagen type IV, and 10 µg/mL LN511F. Measurement of TEER values of iEPCs over a long period (from day 2 to 10) and the maximum value among them is shown. Data are presented as the mean ± SD (*n* = 3; $^{**}p < 0.01$; Tukey's HSD test; 1 × 10^5 group vs. others).

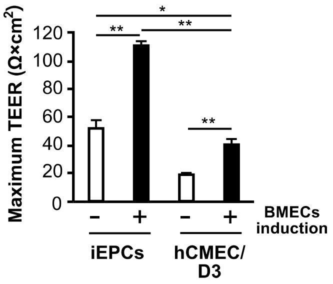

Figure S3. TEER values of hCMEC/D3 cultured under BMEC-inducing conditions

Thawed iEPCs or hCMEC/D3 cells (1 × 10^5 cells/insert) were cultured under the same conditions as Fig. 3A and B. Measurement of TEER values from day 2 to 10 and the maximum value among them is shown. Data are presented as the mean ± SD (*n* = 3; $^{*}p < 0.05$, $^{**}p < 0.01$; Tukey's HSD test).
